# A New Criterion Construction and Verification for GNSS Satellite Selection Based on Near-Real-Time Accuracy

**DOI:** 10.3390/s25237218

**Published:** 2025-11-26

**Authors:** Yue Zuo, Yibin Yao, Mingxian Hu

**Affiliations:** School of Geodesy and Geomatics, Wuhan University, Wuhan 430079, China

**Keywords:** GNSS, satellite selection, near-real-time accuracy, WGDOP

## Abstract

Global Navigation Satellite Systems (GNSS) have undergone more than half a century of development and construction, with more than a hundred navigation satellites currently providing precise and reliable positioning, navigation, and timing (PNT) services for various users. Meanwhile, efficient utilization of these satellites has become a topic of interest. Selecting an appropriate satellite set in a proper manner can reduce computational burden while ensuring positioning accuracy. Geometric Dilution of Precision (GDOP) is commonly used in satellite selection as it quantifies the impact of satellite geometry on positioning accuracy. Due to its computational simplicity, GDOP has been widely applied in satellite selection, but it only considers the satellite geometric configuration while ignoring the quality of satellite observations. As a result, the selected satellite set may lead to poor positioning accuracy. To address this issue, we use a satellite selection criterion based on the combination of near-real-time accuracy of satellite observations and geometric configuration. This criterion utilizes the combination of Geometry-Free Ionosphere-Free (GFIF) and Melbourne–Wübbena (MW) linear combinations of observations. Through a sliding window, we estimate the near-real-time accuracy of observations and use it to calculate the Weighted Geometric Dilution of Precision (WGDOP) for satellite selection. In a global International GNSS Service (IGS) station validation experiment, the satellite set selected based on WGDOP using near-real-time accuracy of GFIF and MW observations improved overall positioning accuracy by 11.6% and 12% when compared with the GDOP-based selection, and by 6% and 6.4% when compared with the Signal-to-Noise Ratio (SNR) weighting method. In a low-cost device validation experiment, the satellite selection method based on near-real-time accuracy of GFIF and MW improved positioning accuracy by 22.5% and 19.7% when compared with the GDOP-based method, and by 23.3% and 20.5% when compared with the SNR-based method. A set of dynamic observation experiments further demonstrates that the satellite selection method based on the near-real-time accuracy of GFIF and MW combinations outperforms the other two selection criteria in dynamic scenarios.

## 1. Introduction

Since its inception, satellite navigation and positioning technology have played a vital role in social and economic development, becoming a key focus area for competition among nations. Currently, the major satellite navigation systems established or under construction globally include the United States’ GPS, Russia’s GLONASS, the European Union’s Galileo, China’s BeiDou Navigation Satellite System (BDS), Japan’s Quasi-Zenith Satellite System (QZSS), and India’s Regional Navigation Satellite System (IRNSS). The abundance of navigation satellite resources has resulted in a higher number of available satellites and greater redundancy, which enhances observational information and improves positioning accuracy. However, excessive redundant observational data can place a computational burden on user terminal devices, increasing the complexity and resource consumption of real-time positioning calculations. Therefore, in practical applications, it is necessary to screen visible navigation satellites and construct an optimal satellite subset to optimize positioning solutions.

The primary factors influencing user positioning accuracy include the precision of satellite observations and the geometric configuration of the satellite subset used in the solution. It is generally accepted that among several satellite subsets, those with smaller GDOP values are more likely to achieve higher positioning accuracy. Minimizing GDOP is considered by some studies to be the preferred satellite selection method in practical applications. Ref. [1] analyzed the minimum GDOP value for five satellites in dual-system configurations and proposed a rapid GDOP computation method. Other research has focused on finding the satellite subset that maximizes the volume of the polyhedron formed by the endpoints of user-satellite unit direction vectors, aiming to achieve the minimum GDOP [2,3,4]. However, subsequent research revealed that the relationship between GDOP and the volume of the polyhedron formed by user-satellite unit vectors does not guarantee that maximizing the volume yields the optimal GDOP value. When considering satellite distribution, a geometric configuration closer to a regular polyhedron should also be prioritized [5].

Current research largely focuses on rapidly identifying satellite subsets corresponding to the minimum GDOP using various methods. Ref. [6] introduced an improved particle swarm optimization algorithm (IPSO) for the rapid selection of satellites in multi-GNSS maritime positioning. Ref. [7] proposed an optimal satellite selection model for global navigation satellite systems (GNSS) based on a genetic algorithm (GA). Ref. [8] analyzed the temporal correlation of satellite selection results and proposed a fast satellite selection algorithm based on an improved beetle antenna search (MBAS). Ref. [9] presented an unsupervised learning-based satellite selection algorithm (ULiSeS), validated through simulations of GPS and NavIC constellations, demonstrating its effectiveness. Some researchers have applied machine learning techniques to satellite selection. Ref. [10] developed an end-to-end satellite selection deep learning network based on PointNet and VoxelNet to address the satellite selection problem. Based on that, ref. [11] used Doppler and acquisition peak metrics as inputs, achieving improved results. Ref. [12] proposed a method for achieving smaller GDOP values based on the Sherman–Morrison formula. This approach dynamically handles increasing numbers of visible satellites and identifies satellite subsets with GDOP values below a specified threshold without predefining the number of satellites to be selected.

In addition to the geometric configuration of satellite subsets, the quality of satellite observations is also a crucial factor influencing the final positioning results. Some existing approaches improve positioning performance by assessing the quality of satellite observations. The WAAS (Wide Area Augmentation System) method evaluates user observation quality using satellite error components estimated from external reference stations, and it excludes low-quality satellites from the visible set [13]. RAIM (Receiver Autonomous Integrity Monitoring), on the other hand, performs a consistency check on the post-solution residuals and removes satellites identified as faulty [14,15]. These methods primarily focus on eliminating low-accuracy or faulty satellites rather than the prior selection of visible satellites. Therefore, they can be applied after the satellite selection. Some studies have employed weighted least squares, using empirical formulas to calculate the overall error of satellites as the basis for weighting [16,17]. Ref. [18] determined weights using a random model based on elevation angles, and experimental results demonstrated that WDOP more accurately reflected the trends in positioning errors compared to conventional DOP. Ref. [19] applied this approach to satellite selection for high-orbit spacecraft and achieved notable results. Researchers have also utilized SNR-weighted WGDOP as a criterion for satellite selection, showing that satellites selected based on this metric provide higher positioning accuracy [20,21]. In this study, we propose a satellite selection method that integrates near-real-time precision and satellite geometry. The near-real-time accuracy is estimated using a sliding window to characterize the quality of the GNSS observations. This estimated accuracy is then used to derive the observation weights, which are subsequently incorporated into the computation of WGDOP for satellite selection. The proposed method is validated using globally distributed IGS stations and real-world measurement data.

The following sections discuss the WGDOP calculation method and introduce the estimation method of near-real-time accuracy of satellite pseudorange observations. Through various experiments, the paper verifies the superiority of this criterion over traditional GDOP and WGDOP satellite selection based on SNR weighting. Finally, conclusions are drawn based on the findings.

## 2. Calculation of WGDOP

This section primarily introduces the GNSS pseudorange observation model and discusses the calculation method of WGDOP based on weighted least squares.

### 2.1. GNSS Pseudorange Observation Model

The GNSS pseudorange observation equation is expressed as follows:

(1) $$P=\rho +c(\delta t_{i}-\delta t^{s})+I_{i}^{s}+T_{i}^{s}+M+\varepsilon$$

where P is the pseudorange observation value, ρ is the distance between the receiver and satellite, c is the speed of light, $\delta t_{i}$ is the receiver clock bias, $\delta t^{s}$ is the satellite clock bias, $I_{i}^{s}$ is the ionospheric delay, $T_{i}^{s}$ is the tropospheric delay, M is the multipath effect error, and ε is the pseudorange observation noise.

In this equation, the receiver position coordinates and clock bias are treated as unknowns for estimation. Satellite clock bias can be corrected based on the ephemeris. Ionospheric delay can be corrected by the broadcast ionosphere model. Tropospheric delay can be mitigated using Saastamoinen’s model. Errors like multipath effects and observation noise, which are not modeled, are challenging to correct. In real-time positioning, these errors are often mitigated by selecting a better observation environment or using specially designed antennas [22]. Assuming other errors are corrected, in the case of single-system (i.e., only GPS, BeiDou, GALILEO et al.) positioning, the linearized model is given by the following equation:

(2) y=Gx

where $y=P-\rho_{0}-D$, $\rho_{0}$ is the initial distance, D includes corrections for satellite clock bias and atmospheric delays, $x={\left[\delta x\;\delta y\;\delta z\;c\delta t_{i}\right]}^{T}$, x includes receiver position coordinates, and clock bias information, and G is as defined in Equation (3). Using the least squares method, the unknowns in Equation (2) can be estimated as follows:

(3) $$G=\left[\begin{array}{cccc} e_{x}^{1} & e_{y}^{1} & e_{z}^{1} & 1\\ \vdots & \vdots & \vdots & \vdots \\ e_{x}^{n} & e_{y}^{n} & e_{z}^{n} & 1 \end{array}\right]$$

(4) $$\hat{x}={\left(G^{T}G\right)}^{-1}G^{T}y$$

where e is the unit vector in the direction from the receiver to the corresponding satellite. In the case of multi-GNSS position, the number of elements in the parameter vector x increases, and consequently, the G matrix needs to be expanded.

### 2.2. Process of Derivation

Considering the varying quality of observations from different satellites, different weight values are calculated based on the magnitude of errors contained in each satellite observation. Subsequently, a weighted least squares method is applied to calculate the receiver coordinate and clock bias. Building upon Equation (4), the solution for the weighted least squares is given as follows:

(5) $${\hat{x}}_{w}={\left(G^{T}WG\right)}^{-1}G^{T}Wy$$

where ${\hat{x}}_{w}$ is the solution of weighted least squares, the unknowns are the same as those in $\;\hat{x}$, and W represents the observation weight matrix. W can be expressed as follows:

(6) $$W=\left[\begin{array}{cccc} w_{1} & 0 & \ldots & 0\\ 0 & w_{2} & \ldots & 0\\ \vdots & \vdots & \ddots & \vdots \\ 0 & 0 & \ldots & w_{n} \end{array}\right]$$

where w represents the weights of different satellite observations.

The covariance matrix of $\hat{x}$ can be estimated using Equation (4). Assuming that all observations have the same precision $\sigma_{0}^{2}$, the observation variance–covariance matrix becomes $\sigma_{0}^{2}I$, where E[*] denotes the expectation operator, COV[*] denotes the variance–covariance operator, and I is the identity matrix. Under this assumption, we obtain the following equation:

(7) $$\begin{array}{cc} COV[\hat{x}] & =E[\hat{x}{\hat{x}}^{T}]\\ & =E[{\left(G^{T}G\right)}^{-1}G^{T}yy^{T}G{\left(G^{T}G\right)}^{-1}]\\ & ={\left(G^{T}G\right)}^{-1}G^{T}E[yy^{T}]G{\left(G^{T}G\right)}^{-1}\\ & ={\left(G^{T}G\right)}^{-1}G^{T}\sigma_{0}^{2}IG{\left(G^{T}G\right)}^{-1}\\ & =\sigma_{0}^{2}{\left(G^{T}G\right)}^{-1} \end{array}$$

Similarly, based on Equation (5), we can obtain the following equation:

(8) $$COV[{\hat{x}}_{w}]=\sigma_{0}^{2}{\left(G^{T}WG\right)}^{-1}$$

The elements on the diagonal of the covariance matrix reflect the magnitude of the a priori errors, thus allowing the derivation of methods for calculating GDOP and WGDOP. Generally, a smaller value of these values is considered indicative of higher positioning accuracy.

(9) $$\begin{array}{c} GDOP=\sqrt{Trace({\left(G^{T}G\right)}^{-1})}\\ WGDOP=\sqrt{Trace({\left(G^{T}WG\right)}^{-1})} \end{array}$$

The reduction in GDOP generally implies a decrease in the estimation error of the position. However, this criterion only considers the geometric configuration of the satellite set. In the case of poor observation quality of part of the satellite set, having the best geometric configuration of the satellite set may not bring the most accurate positioning result. Therefore, we aim to calculate WGDOP, a new criterion for satellite selection, by appropriately setting weights while simultaneously considering the geometric configuration of the satellite set and the quality of observations.

## 3. Construction of the Criterion

In this section, we will process the combination of Geometry-Free Ionosphere-Free (GFIF) and Melbourne–Wübbena (MW) observations. Subsequently, we will introduce the method for estimating near-real-time accuracy and explain the approach for determining weights based on this value.

### 3.1. The Residual Term of the Combination of GFIF and MW

The MW combination [23,24] is obtained by subtracting the narrow-lane combination of pseudorange observations from the wide-lane combination of phase observations in the same epoch. This combination eliminates terms such as geometric distance, ionospheric delay, tropospheric delay, and clock bias. The remaining terms mainly include wide-lane ambiguity, multipath effects, hardware delays, and observation noise. The MW combination is expressed as follows:

(10) $$\begin{array}{cc} MW & =\frac{f_{1}L_{1}-f_{2}L_{2}}{f_{1}-f_{2}}-\frac{f_{1}P_{1}+f_{2}P_{2}}{f_{1}+f_{2}}\\ & =b_{WL}+B_{NL}+N_{WL}+m_{WL}+M_{NL}+\varepsilon_{WL}+E_{NL} \end{array}$$

where L represents the carrier phase observations, P represents the pseudorange observations, f is the corresponding frequency, $b_{WL}$ is the hardware delay bias for the wide-lane carrier phase observations, $B_{NL}$ is the hardware delay bias for the narrow-lane pseudorange observations, $N_{WL}$ is the wide-lane ambiguity, $m_{WL}$ is the multipath effect on the wide-lane carrier phase observations, $M_{NL}$ is the multipath effect on the narrow-lane pseudorange observations, $\varepsilon_{WL}$ is the observation noise of the wide-lane carrier phase observations, and $E_{NL}$ is the observation noise of the narrow-lane pseudorange observations. Given the orders-of-magnitude difference in the correlated error terms between carrier-phase and pseudorange observations, we distinguish these two components at this point.

By differencing the MW combination observations between epochs, in the absence of cycle slips, the wide-lane ambiguity parameters can be eliminated. Additionally, existing studies have indicated that the hardware delay bias does not change significantly in a very short time [25,26]. Therefore, it can be considered that the inter-epoch difference also eliminates this item, and the inter-epoch difference between i and i + 1 can be expressed as follows:

(11) $$\Delta MW^{i,i+1}=\Delta m_{WL}^{i,i+1}+\Delta M_{NL}^{i,i+1}+\varepsilon_{WL}^{i,i+1}+E_{NL}^{i,i+1}$$

At this point, the right side of the equation contains only terms related to multipath effects and observation noise. Considering that the multipath effects and observation noise associated with the carrier phase are much smaller than those associated with the pseudorange, even after amplification and scaling of the wide-lane and narrow-lane observations, the error terms related to the pseudorange can still be dozens of times larger than those related to the carrier phase [27,28,29]. Therefore, the terms related to the carrier phase can be neglected. Hence, the equation is only associated with terms related to the pseudorange, as shown below.

(12) $$\begin{array}{c} \varepsilon_{WL}^{i,i+1}+E_{NL}^{i,i+1}\approx E_{NL}^{i,i+1}\\ \Delta m_{WL}^{i,i+1}+\Delta M_{NL}^{i,i+1}\approx \Delta M_{NL}^{i,i+1} \end{array}$$

Equation (11) can be approximated as follows:

(13) $$\Delta MW^{i,i+1}\approx \Delta M_{NL}^{i,i+1}+E_{NL}^{i,i+1}$$

The GFIF combination [30,31] can be used to calculate the pseudorange multipath and is applied in the internationally renowned satellite observation file quality checking tool TEQC. This linear combination, taking L1 as an example, is expressed as follows:

(14) $$\begin{array}{ll} GFIF_{1} & =P_{1}-\frac{\alpha +1}{\alpha -1}L_{1}+\frac{2}{\alpha -1}L_{2}\\ & =M_{1}+B_{1}+N_{I}-\frac{\alpha +1}{\alpha -1}(m_{1}+b_{1})+\frac{2}{\alpha -1}(m_{2}+b_{2})+E_{P}+\varepsilon_{L} \end{array}$$

where $N_{I}$ represents the ${GFIF}_{1}$ ambiguity combination, and α is the ionosphere elimination coefficient. These two terms are expressed as follows:

(15) $$N_{I}=-\frac{\alpha +1}{\alpha -1}N_{1}\lambda_{1}+\frac{2}{\alpha -1}N_{2}\lambda_{2}$$

(16) $$\alpha =\frac{f_{1}^{2}}{f_{2}^{2}}$$

where N represents the ambiguity parameter, and λ is the wavelength.

Referring to the processing method for the MW combination mentioned earlier, by differencing between epochs to eliminate ambiguity parameters and hardware delays, and neglecting the carrier phase multipath effects and observation noise, the final equation can be written as follows:

(17) $$\Delta GFIF_{1}^{i,i+1}\approx \Delta M_{1}^{i,i+1}+E_{P}^{i,i+1}$$

As shown in Equations (13) and (17), the terms ${\Delta MW}^{i,i+1}$ and ${\Delta GFIF}_{1}^{i,i+1}$, derived from the MW and GFIF combinations, include only the components related to pseudorange multipath and pseudorange observation noise. Both of which are crucial errors affecting positioning accuracy.

### 3.2. Near Real-Time Accuracy Estimation and Weight Determination

**Figure 1:**
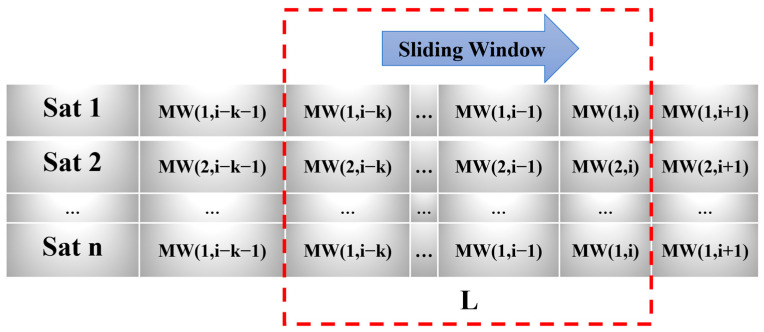
Schematic diagram of the sliding window.

Since the residual terms are important sources of errors affecting positioning and change slowly, the accuracy information can reflect the quality of satellite pseudo-range observations by calculating and estimating multiple epoch combinations in a short period of time in real-time positioning. By setting a sliding window and calculating using a few epochs with a short time interval from the current epoch, the dynamically estimated near-real-time accuracy for the current epoch can be obtained. Taking the calculation results of the MW combination as an example, the setting of the sliding window is illustrated in Figure 1.

For the current epoch i, with a sliding window of length L, the combination observations for L epochs corresponding to the satellite are calculated. And L-1 differential results are generated. According to Equation (18), the differences between L epochs are estimated to determine the near-real-time accuracy $\sigma_{n}^{i}$ of the current epoch i for satellite n. The process of estimating the near-real-time accuracy for the current epoch corresponding to the satellite using the GFIF combination is the same as that for the MW combination. It should be noted that detecting small error perturbations typically requires high-frequency GNSS receivers [32,33]. However, as long as the unmodeled errors in consecutive epochs—such as multipath effects—exhibit significant variations, these relative changes are reflected in the resulting near-real-time accuracy. Consequently, the computed accuracy effectively represents the quality of satellite observations over the recent short time interval.

(18) $$\sigma_{n}^{i}=\sqrt{\frac{\sum_{k=i-L+1}^{L-1}{\left[MW(n,k+1)-MW(n,k)\right]}^{2}}{2(L-1)}}$$

The observation data of IGS station CUT0 (32°0′ N, 115°53′ E) UTC 2023001 throughout the day were selected with a time interval of 30 s. In previous studies on GNSS observation precision, short-span GNSS observations are generally used to estimate observation accuracy and to fit empirical models [28,29]. Considering the time spans adopted in these historical studies, the sliding window in this work is set to a duration of 210 s. Based on the receiver sampling rate, the corresponding sliding window length *L* is calculated as follows:

(19) $$L=\frac{dT}{sample}+1$$

where *dT* is the time span of the sliding window, and *sample* denotes the receiver sampling interval.

**Figure 2:**
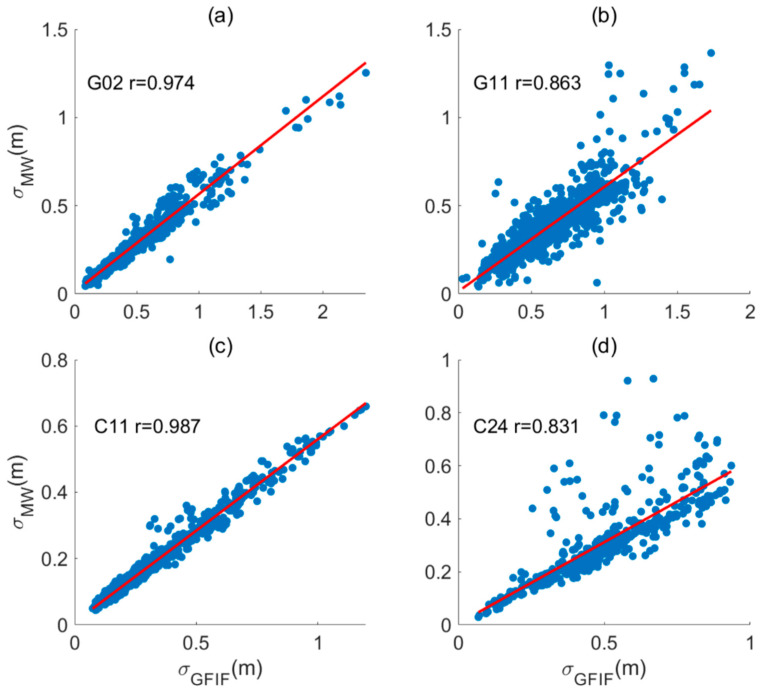
Correlation analysis of near-real-time accuracy derived based on Geometry-Free Ionosphere-Free (GFIF) and Melbourne–Wübbena (MW) observations. (**a**) Satellites with the maximum correlation coefficient in the GPS system. (**b**) Satellites with the minimum correlation coefficient in the GPS system. (**c**) Satellites with the maximum correlation coefficient in the BDS system. (**d**) Satellites with the minimum correlation coefficient in the BDS system.

The near-real-time accuracy sequences of GFIF and MW are estimated, and correlation analysis is performed. The correlation analysis results for some satellites are shown in Figure 2. For GPS, G02 has the highest correlation coefficient, which is 0.974, and G11 has the lowest, which is 0.863. For BDS, C11 and C24 are the satellites with the highest and lowest correlation coefficients, which are 0.987 and 0.831, respectively. It can be observed that the two sequences have a high correlation. This is because the residual errors from both calculation methods come from multipath errors and the noise. The highly similar contents of residual terms also indicate that the multipath and observation noise trends in pseudorange observations at different frequencies are similar.

**Figure 3:**
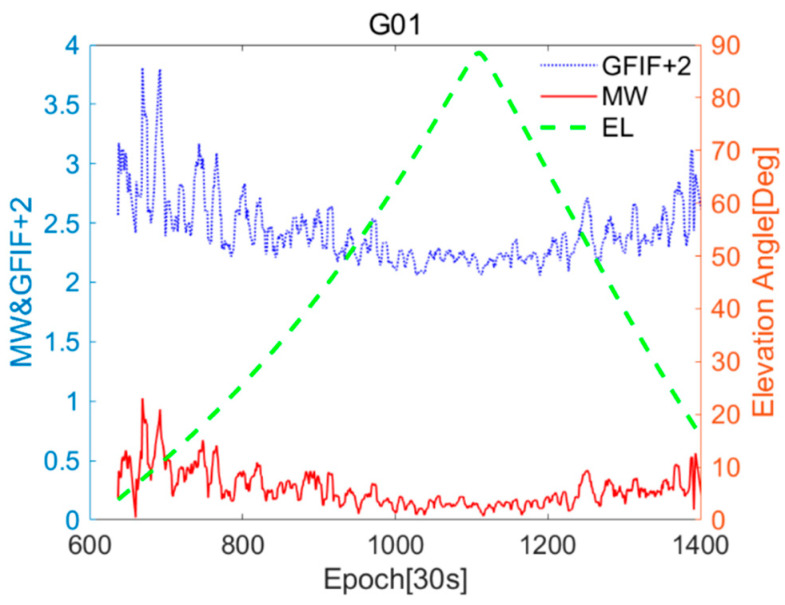
CUT0 station G01 of Geometry-Free Ionosphere-Free (GFIF) and Melbourne–Wübbena (MW) near-real-time accuracy sequences and elevation angle changes.

**Figure 4:**
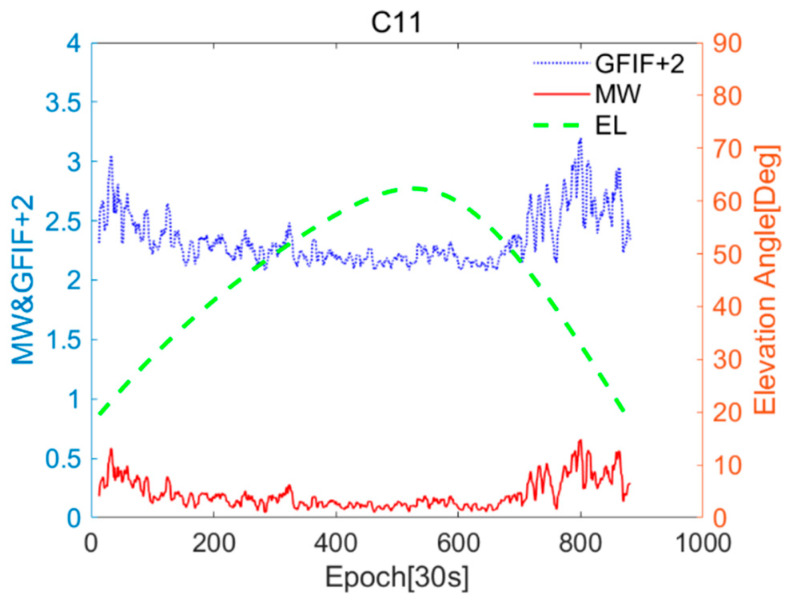
CUT0 station C11 of Geometry-Free Ionosphere-Free (GFIF) and Melbourne–Wübbena (MW) near-real-time accuracy sequences and elevation angle changes.

The relationship between the near-real-time accuracy sequences of GFIF and MW for G01 and C11, and the changes in the elevation angle are shown in Figure 3 and Figure 4. The GFIF sequence is uniformly increased by 2 for better differentiation. When the satellite elevation angle is high, the values of the GFIF and MW residual terms for the satellite are generally small. As the elevation angle gradually decreases, these values tend to increase and become unstable. This trend is consistent with the variation in multipath errors and observation noise with the elevation angle [29].

According to Equation (18), the weight matrix of the pseudorange observations for n satellites in epoch i can be expressed as follows:

(20) $$W=\left[\begin{array}{cccc} {\left(\frac{\sigma_{0}}{\sigma_{1}^{i}}\right)}^{2} & 0 & \ldots & 0\\ 0 & {\left(\frac{\sigma_{0}}{\sigma_{2}^{i}}\right)}^{2} & \ldots & 0\\ \vdots & \vdots & \ddots & \vdots \\ 0 & 0 & \ldots & {\left(\frac{\sigma_{0}}{\sigma_{n}^{i}}\right)}^{2} \end{array}\right]$$

The unit weight variance of satellite observations is denoted by $\sigma_{0}$, and its specific value can be set accordingly. Subsequently, using Equation (9), the GDOP and WGDOP values for the corresponding set of satellites can be calculated.

### 3.3. The Process of Satellite Selection

**Figure 5:**
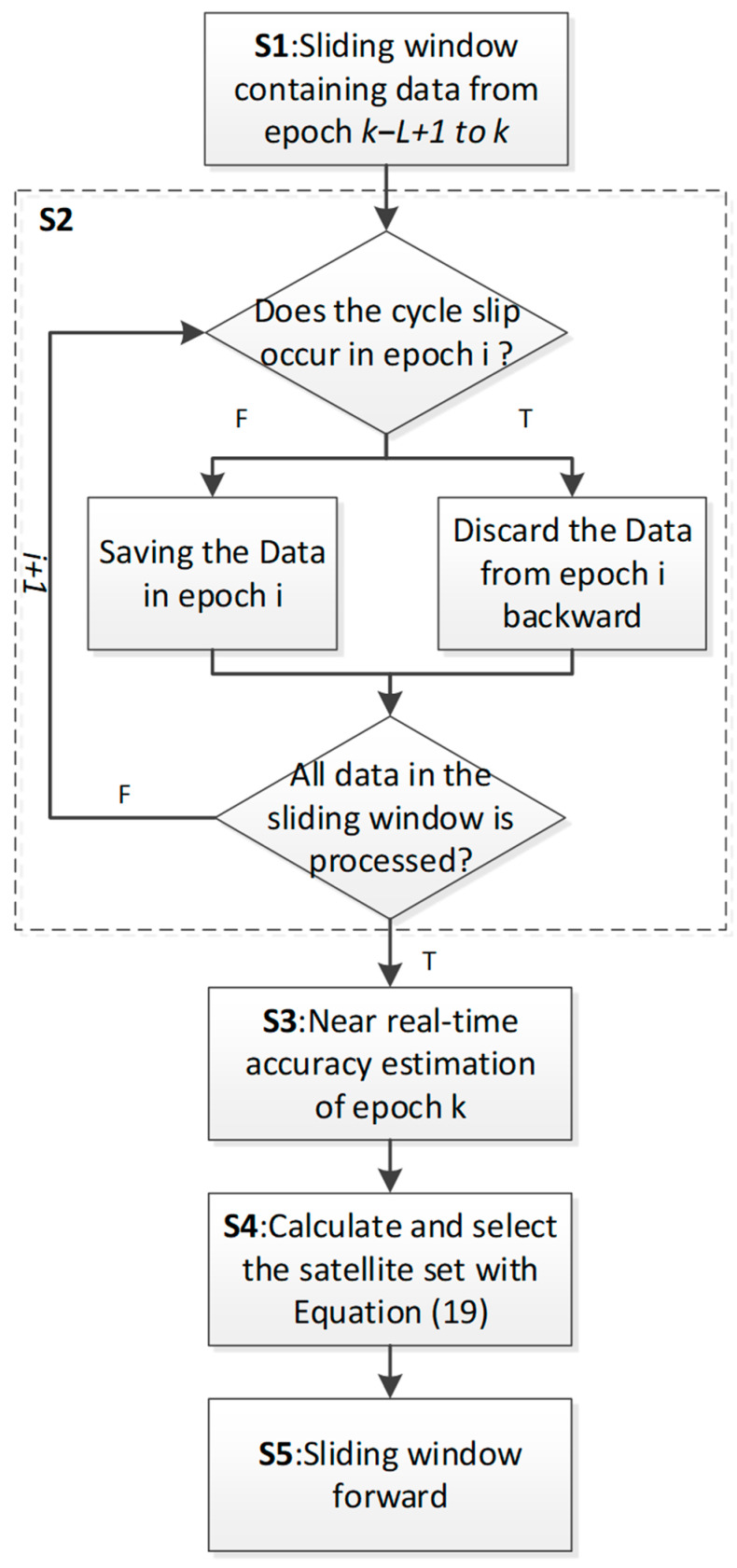
The process of satellite selection.

Based on the calculation method of the satellite observation weight matrix described in the previous two sections, the process of satellite selection used in this paper is shown in Figure 5. The specific steps can be expressed as follows.

**S1:** Select visible satellite data from i − L + 1 to i epoch in the sliding window.

**S2:** Starting from epoch i − L + 1 and ending in epoch i, perform cycle slip detection for each satellite and epoch. Data before the epoch with the cycle slip for each satellite will be discarded (details are shown in the dashed box in Figure 5).

**S3:** Estimate the near-real-time accuracy for epoch i based on Equation (18).

**S4:** Calculate the WGDOP for each satellite set and select the set with the minimum WGDOP. Various satellite selection methods can be employed in this step. To validate the reasonability of WGDOP, this paper uses a brute force method [10] to accurately select the satellite set with the minimum WGDOP.

**S5:** Move the sliding window forward and repeat steps **S1**–**S4**.

Through this process, a satellite set with the minimum WGDOP based on near-real-time accuracy can be computed for each epoch. In comparison to GDOP-based satellite selection, this method considers both satellite geometry and the quality of satellite observations.

## 4. Experimental Results and Analysis

In this section, we will use GDOP and WGDOP as criteria for satellite selection and employ a brute force method to unbiasedly select the optimal satellite set corresponding to each criterion. The near-real-time accuracy weighted satellite selection method based on the combinations of GFIF and MW will be compared with methods based on SNR weighting or without weighting (based on GDOP). The SNR weighting method used by [21] is employed, and the positioning accuracy of different satellite sets selected by different methods will be compared.

To evaluate the performance of different satellite selection criteria, we utilized data from global IGS stations, the measured data from a low-cost receiver, and a set of dynamic GNSS observation data. Data from IGS stations cover the period from UTC 2023001 to 2023007, the low-cost receiver data were taken in UTC 2023296, and the set of dynamic GNSS observation data was collected in UTC 2023012.

### 4.1. Validation of IGS Station Data

**Figure 6:**
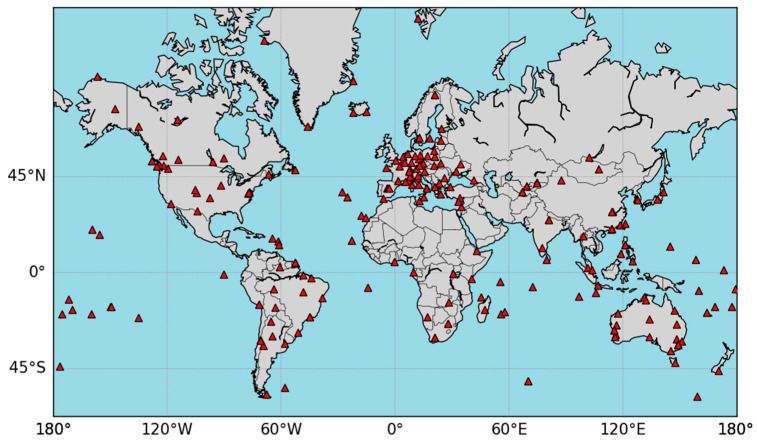
Distribution of selected IGS stations.

The global distribution of selected IGS stations is illustrated in Figure 6, with a total of 235 stations. The observation period spans from UTC 2023001 to 2023007, and the observation data interval is 30 s. Due to the high correlation between neighboring epochs, the optimal satellite sets selected are highly likely to be the same [8]. In this experiment, the sampling interval is set to 10 min (144 epochs in a day), and a longer observation period is chosen to diversify the data state of the satellite sets. The estimation of near-real-time accuracy is still performed at 30-s intervals. Eight GPS + BDS dual-system medium earth orbit (MEO) satellites are selected, with a sliding window length of 8, a cutoff elevation angle uniformly set to 10°, and a unit weight variance for near-real-time accuracy estimation set at 1.0 m.

Using the single point positioning (SPP) method, we conducted positioning using GPS + BDS pseudorange observations. Broadcast ephemeris was utilized for satellite orbit information, and ionospheric corrections were performed using the broadcast ionosphere model. Tropospheric corrections were applied using Saastamoinen’s model. The station coordinates with millimeter-level accuracy in the weekly SNX file published by IGS are selected as the true values to calculate the positioning accuracy of the satellite set selected by different satellite selection criteria. The calculation of satellite positioning accuracy is expressed as follows:

(21) $$\sigma^{2}=\frac{\sum_{i=1}^{N}\left[{\left(X_{i}-X_{0}\right)}^{2}+{\left(Y_{i}-Y_{0}\right)}^{2}+{\left(Z_{i}-Z_{0}\right)}^{2}\right]}{N}$$

where σ is the positioning accuracy of the site, $\left[X_{i}Y_{i}Z_{i}\right]$ is the positioning result of epoch i, $\left[X_{0}Y_{0}Z_{0}\right]$ is the true value of the coordinates of the station, and N is the number of epochs.

**Figure 7:**
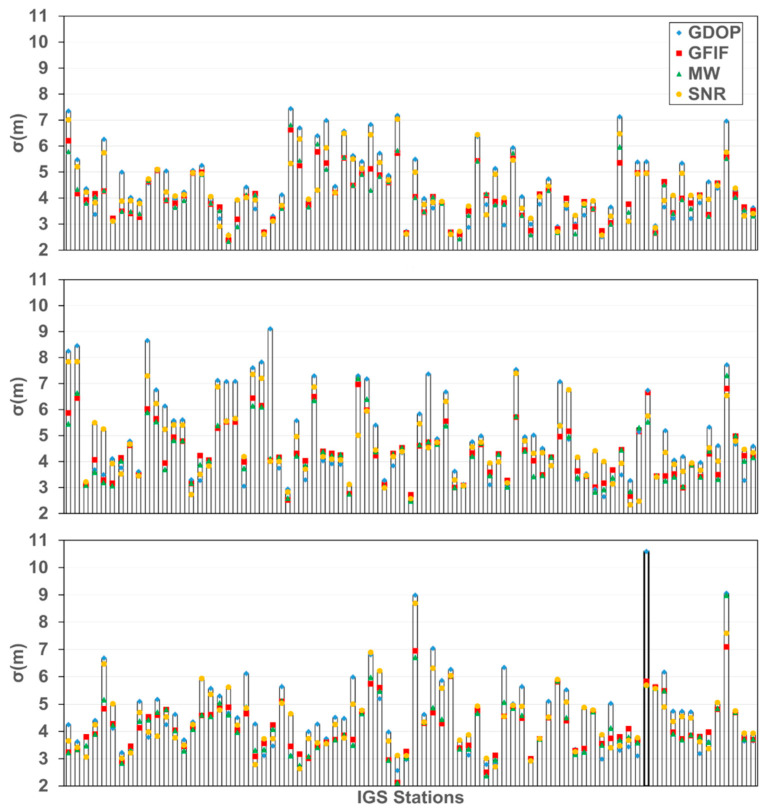
Positioning accuracy of different satellite selection criteria at 235 IGS stations.

The overview of station positioning accuracy is shown in Figure 7, which includes results for all 235 stations. From the figure, it can be observed that, for nearly 78.3% of the stations, the positioning accuracy based on the satellite set selected using WGDOP is better than that based on the set selected using GDOP. As highlighted in the bolded section of the figure, ignoring observation quality may result in significant positioning errors when using GDOP. In contrast, the new indicator can effectively mitigate such issues. However, there are cases where the opposite is true. The main reason is that the weighting is based on the near-real-time precision estimation using the observation value sequence before the current epoch. This performs well when the receiver observation noise and multipath variation are relatively stable over a short period. Nevertheless, this method cannot predict the future epoch’s observation error changes. In situations of drastic changes in observation errors, achieving good positioning results becomes challenging. Additionally, when there is data loss in the sliding window, such as signal loss, cycle slips, or low satellite elevation angles, there is insufficient data to accurately estimate the near-real-time precision.

The general positioning accuracy statistics are presented in Table 1. Four satellite selection methods are considered: GDOP-based selection, GFIF near-real-time precision weighting selection, MW near-real-time precision weighting selection, and SNR-based selection, denoted as Method 1 to Method 4, respectively. The SPP accuracy for Methods 1 to 4 is 4.875 m, 4.308 m, 4.290 m, and 4.583 m, respectively. Method 2 shows an improvement of 11.6% and 6% in accuracy when compared to Methods 1 and 4, respectively. Method 3 demonstrates improvements of 12% and 6.4% when compared to Methods 1 and 4, respectively.

The comparison of positioning performance among methods is shown in Table 2. For Method 2–4, among the 235 stations, approximately 68.5%, 71.9%, and 68.1% of the stations show improved positioning accuracy when compared to those with Method 1. Results of Method 3 outperform those of other selection methods in both accuracy and proportion. Method 2 shows that the accuracy is nearly identical to that of Method 3, but with a lower proportion, slightly lagging behind Method 3, and outperforming the other two methods. The above results preliminarily demonstrate the reliability of the new criteria.

**Figure 8:**
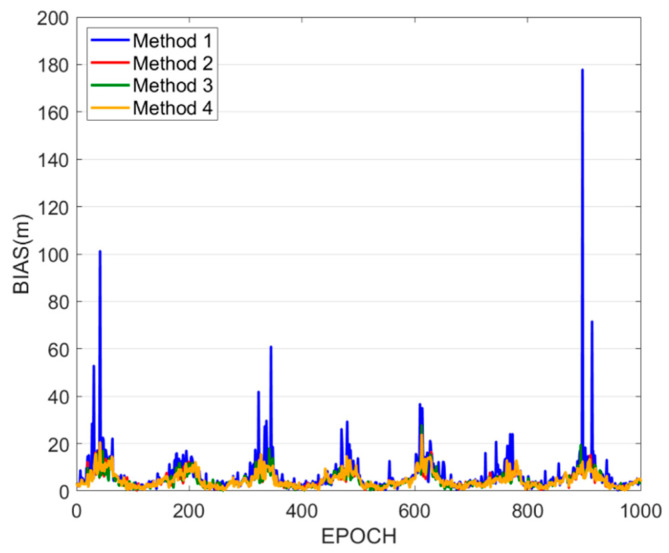
Method 1–4 positioning bias sequence for the WUH2 station.

For those stations with abnormal results in the above findings, this study selected WUH2, which is bold in Figure 7, for further analysis. The positioning bias sequences for Methods 1–4 at WUH2 are presented in Figure 8, with positioning accuracies of 10.60 m, 5.83 m, 5.69 m, and 5.69 m, respectively. Method 1 shows significantly larger bias in certain epochs when compared to other methods, severely impacting the station’s positioning accuracy. Therefore, the selection of satellite sets should also comprehensively consider factors related to the quality of observations.

### 4.2. Validation of Data from a Low-Cost Receiver

**Figure 9:**
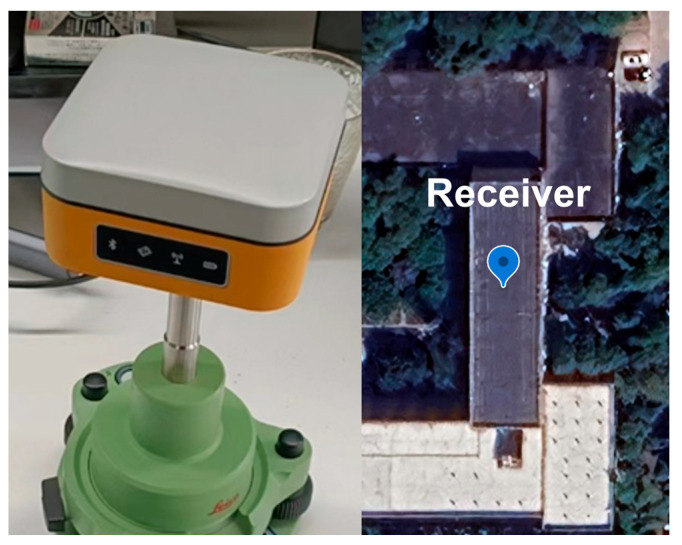
The low-cost receiver and its deployment location.

To further validate the application effectiveness of the new criterion in the low-cost receiver, this study utilized a low-cost positioning receiver priced at around two hundred USD. The receiver is based on the Unicore UM960 positioning module and is integrated into a Linux + Qualcomm MDM9628 Cortex-A7 platform. It was deployed at a known point on the rooftop of the School of Geodesy and Geomatics at Wuhan University (30°31′ N, 114°21′ E). The schematic diagram of the receiver and its deployment location is illustrated in Figure 9. The receiver can collect three-frequency data from GPS + BDS. The receiver’s sampling rate is set to 1 Hz, and the sampling period is from UTC 2023296 4:00 to 5:00. The current sliding window length is set to 211 based on the previous discussion of the time span for the sliding window, and the positioning settings remain consistent with those described earlier.

**Figure 10:**
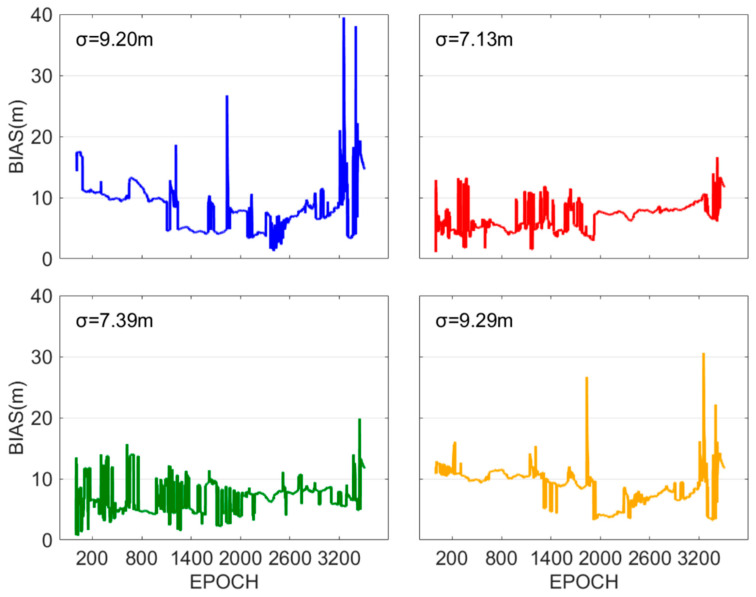
The low-cost receiver positioning bias sequences for Methods 1–4. The blue, red, green, and yellow lines represent the positioning biases sequence of Methods 1–4, respectively.

The positioning bias sequence is illustrated in Figure 10. It can be observed that Methods 2 and 3, based on near-real-time accuracy, show overall smaller positioning biases when compared to Methods 1 and 4. Additionally, the positioning bias sequences of Methods 2 and 3 are more stable, while Methods 1 and 4 show larger fluctuations, with some epochs exhibiting significantly gross errors. The positioning accuracies of Methods 1–4 are 9.20 m, 7.13 m, 7.39 m, and 9.29 m, respectively. Method 2 demonstrates 22.5% and 23.3% improvement in positioning accuracy, whereas Method 3 exhibits 19.7% and 20.5% improvement, both compared to Methods 1 and 4, respectively.

The experimental results indicate that the positioning performance of WGDOP-based satellite selection for near-real-time accuracy is superior to both GDOP-based satellite selection and WGDOP selection based on SNR. Moreover, the WGDOP satellite selection method based on near-real-time accuracy exhibits robustness against gross errors. It is observed that the satellite set with the optimal geometric configuration may not always yield the best positioning results. Additionally, SNR, which primarily characterizes the transmission quality of satellite signals, may not fully reflect the quality of satellite observations. Therefore, the satellite set selected based on SNR might not always result in the best positioning performance.

### 4.3. Validation of Dynamic Data

**Figure 11:**
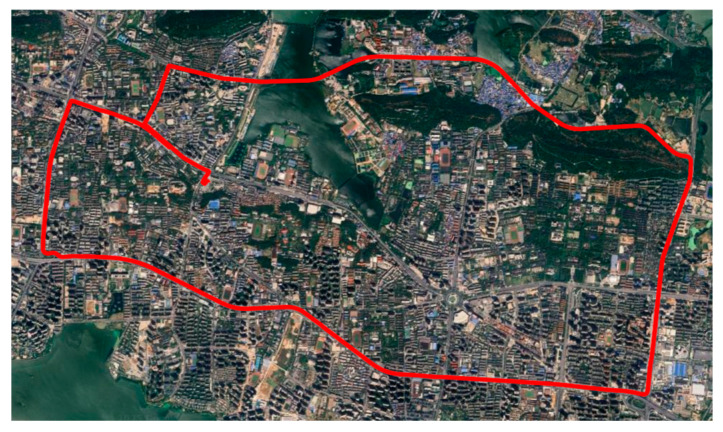
Receiver trajectory in the dynamic experiment conducted in Wuhan, China.

To further validate the effectiveness of the proposed satellite selection criterion, a set of GNSS observation data collected by the UM980 positioning module in a dynamic scenario was used for verification. The receiver sampling rate was set to 1 Hz, and the data collection period was from UTC 2023012 10:50 to 11:40. The experiment utilized multi-GNSS observations from GPS, Galileo, and BDS constellations. The experiment was conducted in Wuhan, Hubei, China, and the receiver trajectory is shown in Figure 11. The sliding window length was set to 211 based on the previous discussion, and the rest of the positioning settings were kept consistent with those described earlier.

**Figure 12:**
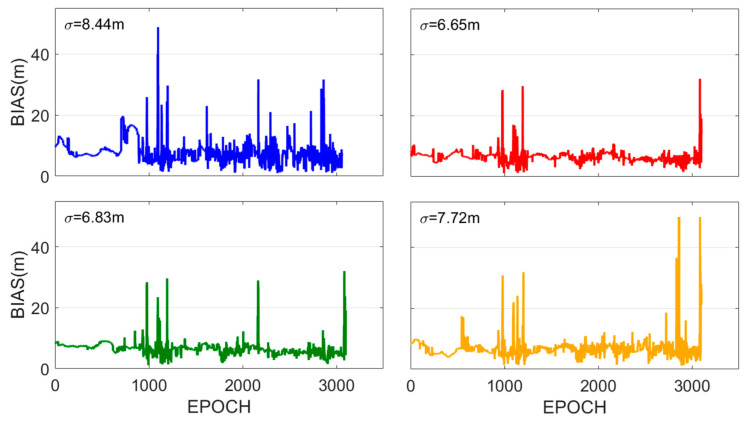
The dynamic data positioning bias sequences for Methods 1–4. The blue, red, green, and yellow lines represent the positioning biases sequence of Methods 1–4, respectively.

The positioning bias series is illustrated in Figure 12. The positioning accuracies of Methods 1–4 are 8.44 m, 6.65 m, 6.83 m, and 7.72 m, respectively. Method 2 demonstrates 21.2% and 13.9% improvements in positioning accuracy, while Method 3 achieves 19.1% and 11.5% improvements compared with Methods 1 and 4, respectively. Consistent with the conclusions drawn from the previous experimental results, the WGDOP-based satellite selection that incorporates near-real-time accuracy outperforms both the GDOP-based selection and the WGDOP selection based on SNR. This further confirms that the proposed satellite selection criterion exhibits superior performance over traditional selection criteria even in dynamic environments.

## 5. Conclusions

Focusing on the insufficient consideration of the quality of satellite observations in current satellite selection research, this paper, based on GFIF and MW combinations, estimates the near-real-time accuracy of satellite observations through a sliding window and constructs the observation weight for calculating WGDOP. This new criterion aims to better balance the geometric configuration of satellite sets and observation quality. Through experiments and numerical analysis, the following conclusions can be drawn:

(1) The near-real-time accuracy sequences estimated based on GFIF and MW combinations exhibit high correlation, attributed to the convergence of residual terms between the two observations. Since near-real-time accuracy primarily encompasses multipath errors and observation noise, its variation is correlated with changes in satellite elevation angles. When the elevation angle is high, the value is relatively small and stable, whereas it gradually increases and becomes unstable as the elevation angle decreases.

(2) Experimental results from 235 IGS stations indicate that approximately 80% of stations benefit from the WGDOP satellite selection method, resulting in improved SPP accuracy for the selected satellite sets. Merely considering the geometric configuration of the satellite set does not lead to accurate positioning results. The overall positioning performance of the WGDOP satellite selection method based on the near-real-time accuracy estimation of GFIF and MW combinations is superior to the positioning performance of the WGDOP satellite selection method based on SNR. The selected satellite set contributes more to enhancing positioning accuracy.

(3) The satellite selection positioning results based on the low-cost device static data and the dynamic data demonstrate that the optimal satellite set selected using a method that integrates near-real-time accuracy and geometric configuration achieves better positioning accuracy. Compared to the other two methods, this approach results in an improvement of approximately 10–20% in positioning accuracy. The experiment also highlights that SNR may not always accurately reflect the quality of satellite observations, and that the satellite set selected based on SNR is not always the optimal choice.

In summary, the proposed satellite selection criterion demonstrates superior performance compared with traditional indicators such as GDOP across different positioning scenarios. Moreover, it has the potential to be combined with advanced satellite selection strategies, including metaheuristic optimization and machine learning methods, to further enhance GNSS positioning performance in applications requiring satellite selection.

## Figures and Tables

**Table 1:** General positioning accuracy of 235 IGS stations.

Method Name	σ (m)
Method 1	4.875
Method 2	4.308
Method 3	4.290
Method 4	4.583

**Table 2:** Pairwise comparison of positioning results among four methods.

Method Pair	Proportion (%)
Method 1/Method 2	31.5:68.5
Method 1/Method 3	28.1:71.9
Method 1/Method 4	31.9:68.1
Method 3/Method 2	61.3:38.7
Method 3/Method 4	65.1:34.9
Method 2/Method 4	63.8:36.2

## Data Availability

Data is contained within the article. The datasets generated and analyzed during the current study are available from the corresponding authors on reasonable request.

## References

[1] Teng Y., Wang J., Huang Q. (2015). Minimum of Geometric Dilution of Precision (GDOP) for five satellites with dual-GNSS constellations. Adv. Space Res..

[2] Blanco-Delgado N., Nunes F.D. (2010). Satellite selection method for multi-constellation GNSS using convex geometry. IEEE Trans. Veh. Technol..

[3] Zhang P., Xu C., Hu C., Chen Y. Research on fast satellite selection algorithm based on geometry. Proceedings of the 4th China Satellite Navigation Conference (CSNC 2013), Lecture Notes in Electrical Engineering.

[4] Kong J., Mao X., Li S. BDS/GPS satellite selection algorithm based on polyhedron volumetric method. Proceedings of the 2014 IEEE/SICE International Symposium on System Integration.

[5] Blanco-Delgado N., Nunes F.D., Seco-Granados G. (2017). On the relation between GDOP and the volume described by the user-to-satellite unit vectors for GNSS positioning. GPS Solut..

[6] Guan X., Chai H., Xiao G., Han J., Han S., Shufeng M. (2021). A fast satellite selection algorithm for multi-GNSS marine positioning based on improved particle swarm optimisation. Surv. Rev..

[7] Zhu S. An optimal satellite selection model of global navigation satellite system based on genetic algorithm. Proceedings of the China Satellite Navigation Conference (CSNC) 2018 Proceedings.

[8] Yu Q., Wang Y., Shen Y. (2022). A fast GNSS satellite selection algorithm for continuous real-time positioning. GPS Solut..

[9] Biswas S.K. (2022). Unsupervised learning-based satellite selection algorithm for GPS–NavIC multi-constellation receivers. GPS Solut..

[10] Huang P., Rizos C., Roberts C. (2018). Satellite selection with an end-to-end deep learning network. GPS Solut..

[11] Singh P., Joshi J., Dey A., Sharma N. (2022). GNSS Satellite Selection-based on Per-satellite Parameters Using Deep Learning. IETE J. Res..

[12] Shi J., Li K., Chai L., Liang L., Tian C., Xu K. (2023). Fast satellite selection algorithm for GNSS multi-system based on Sherman–Morrison formula. GPS Solut..

[13] Proposed Amendment to International Standards and Recommended Practices Aeronautical Telecommunications. Annex 10 to the Convention on International Civil Aviation. Volume I Radio Navigation Aids. https://elibrary.icao.int/product/299828.

[14] Brown G. (1992). A Baseline GPS RAIM Scheme and a Note on the Equivalence of Three RAIM Methods. Navigation.

[15] Diggelen V.F., Brown A. Mathematical aspects of GPS RAIM. Proceedings of the 1994 IEEE Position, Location and Navigation Symposium-PLANS’941994.

[16] Park C., Kim I., Lee J., Jee G. (1996). A satellite selection criterion incorporating the effect of elevation angle in GPS positioning. Control. Eng. Pract..

[17] Sairo H., Akopian D., Takala J. (2003). Weighted dilution of precision as quality measure in satellite positioning. IEE Proc.-Radar Sonar Navig..

[18] Won D.H., Ahn J., Lee S.W., Lee J., Sung S., Park H.W., Park J.P., Lee Y.J. (2012). Weighted dop with consideration on elevation-dependent range errors of GNSS satellites. IEEE Trans. Instrum. Meas..

[19] Wang S., Zhuang X., Shi T., Zeng X., Zeng K. A Method of Satellite Selection for High Orbiter Navigation Based on Equivalent WDOP. Proceedings of the 2023 IEEE International Conference on Unmanned Systems (ICUS).

[20] Li J., Wu M. The Improvement of Positioning Accuracy with Weighted Least Square Based on SNR. Proceedings of the 2009 5th International Conference on Wireless Communications, Networking and Mobile Computing.

[21] Du H., Hong Y., Xia N., Zhang G., Yu Y., Zhang J. (2020). A Navigation Satellites Selection Method Based on ACO With Polarized Feedback. IEEE Access.

[22] Hu M., Yao Y., Ge M., Neitzel F., Shi J., Pan P., Yang M. (2023). Random walk multipath method for Galileo real-time phase multipath mitigation. GPS Solut..

[23] Melbourne W.G. The case for ranging in GPS-based gedetic systems. Proceedings of the First International Symposium on Precise Positioning with the Global Positioning System.

[24] Wübbena G. Software developments for geodetic positioning with GPS using TI-4100 code and carrier measurements. Proceedings of the First International Symposium on Precise Positioning with the Global Positioning System.

[25] Coco D.S., Coker C., Dahlke S.R., Clynch J.R. (1991). Variability of GPS satellite differential group delay biases. IEEE Trans. Aerosp. Electron. Syst..

[26] Wang Y., Feng Y., Zheng F. Geometry-free stochastic analysis of BDS triple frequency signals. Proceedings of the ION ITM 2016, Institute of Navigation.

[27] Zhang Q., Zhang L., Sun A., Meng X., Zhao D., Hancock C. (2024). GNSS Carrier-Phase Multipath Modeling and Correction: A Review and Prospect of Data Processing Methods. Remote Sens..

[28] Li B., Lou L., Shen Y. (2016). GNSS elevation-dependent stochastic modeling and its impacts on the statistic testing. J. Surv. Eng..

[29] Xi R., Meng X., Jiang W., An X., Chen Q. (2018). GPS/GLONASS carrier phase elevation-dependent stochastic modelling estimation and its application in bridge monitoring. Adv. Space Res..

[30] Estey L., Meertens C. (1999). TEQC: The multi-purpose toolkit for GPS/GLONASS data. GPS Solut..

[31] Wang N., Yuan Y., Li Z., Montenbruck O., Tan B. (2016). Determination of differential code biases with multi-GNSS observations. J. Geod..

[32] Demyanov V.V., Danilchuk E.I., Fedorov M.E., Demyanov V. (2025). Modern Improvements of GNSS Technologies: New Opportunities in Exploration of the Earth’s Ionosphere. Satellite Systems for Navigation and Geosciences.

[33] Demyanov V.V., Sergeeva M.A., Yasyukevich A.S., Demyanov V. (2019). GNSS High-Rate Data and Efficiency of Ionospheric Scintillation Indices. Ionospheric and Atmospheric Threats for GNSS and Satellite Telecommunications.

